# First Union Formation in Italy: The Role of Micro- and Macro-Level Economic Conditions

**DOI:** 10.1007/s10680-024-09712-8

**Published:** 2024-11-01

**Authors:** Silvia Meggiolaro, Fausta Ongaro, Elena Pirani

**Affiliations:** 1 Dipartimento di Scienze Statistiche, University of Padua, Padua, Italy; 2 Dipartimento di Statistica, Informatica, Applicazioni – DiSIA, University of Florence, Florence, Italy

**Keywords:** Marriage, Cohabitation, Economic uncertainty, Italy

## Abstract

In this paper, we use data from the ‘Families and Social Subjects’ survey conducted by the Italian National Institute of Statistics in 2016 to study the impact of micro- and macro-level economic conditions on first co-residential union formation. We aim to determine if and to what extent the probability of forming the first union is explained by individual labour market positions (e.g. having non-standard employment or not having work), and additionally explore if adverse macroeconomic conditions also play a role. We differentiate by union type—marriage and cohabitation—known to be characterised by different levels of union commitment. We also address potential gender differences by conducting separate analyses on men and women. Our results suggest that while micro- and macro-level economic factors matter in the union formation process, their effect varies by gender and union type. Individual economic vulnerability has a greater impact on marriage than on cohabitation for both men and women. Instead, contextual economic uncertainty plays a relevant role, especially in the transition to cohabitation, regardless of gender, and, to a lesser extent, in the transition to marriage, but only for women.

## Introduction

Forming a first co-residential union is a crucial step in the transition to adulthood for young people worldwide, and a large body of theoretical and empirical work explores this topic. Numerous studies address the role of economic conditions in this process, where having work and (good) economic prospects are considered a prerequisite for starting an independent family (see, for example, Bukodi, 2012; Jalovaara, 2012; Schneider et al., 2019; Vignoli et al., 2016). This aspect is even more relevant today, in a world marked by labour market complexity (such as the rise in precariousness and unemployment, alongside a decline in secure job positions), economic stagnation (if not recession) and financial turbulence.

However, the association between an individual’s poor economic circumstances and the postponement of co-residential union formation varies by country and gender. In male-breadwinner societies, the male partner’s economic situation tends to be more influential (Raz-Yurovich, 2010). In contrast, in dual-breadwinner settings, women’s economic vulnerability is also relevant (Kreyenfeld et al., 2012). The role of economic conditions in the union formation process may also differ depending on the type of co-residential union. Cohabitation has been observed to exhibit less sensitivity to economic vulnerability than marriage and, as it represents less long-term commitment, is more favoured in the case of poor economic prospects (Kalmijn, 2011; Vignoli et al., 2016).

While individuals’ economic conditions are undoubtedly a fundamental factor in facilitating or obstructing the union formation process, the macro-level context in which they are embedded may also play a role. According to the principle *of time and place* in the framework of life course studies (Elder, 1994), the same historical event may differ in substance and meaning across geographical areas. Accordingly, studying individuals’ outcomes and behaviours must account for contextual influences that operate along temporal and spatial dimensions. For example, Kohler and colleagues (2002) argue that macro-level economic instability leads to micro-level uncertainty, which delays union formation (and childbearing) in favour of prolonged residence in the parental home. This additional time can be used to achieve higher education levels while awaiting greater job stability (see, e.g. Aassve et al., 2007; Kreyenfeld et al., 2012). Young people are particularly vulnerable during economic turbulence, which triggers a state of global uncertainty and undermines long-term planning (see, e.g. Vignoli et al., 2016, 2020). The relatively few empirical studies assessing the impact of individual and aggregate-level economic aspects on the union formation process confirm these effects (De Lange et al., 2014), especially for men (Vergauwen et al., 2016). However, the potentially varying effect of macroeconomic uncertainty on marriage and cohabitation has received little attention.

With this study, we fill an existing gap in the literature by examining whether and to what extent the probability of forming a first co-residential union is solely explained by individual economic vulnerability (e.g. having non-standard employment or not having a job) or if adverse macroeconomic conditions, which may increase individuals’ perception of uncertainty, exert an autonomous effect.

We focus on Italy, an intriguing case study, for several reasons. Demographically, a “revolution” has reshaped the family in recent years. With a several-decade delay compared to continental and Northern European countries, family life courses have begun to relax their rigidity. Alternative strategies—delayed marriage, cohabitation, union dissolution, and out-of-wedlock childbearing—have become increasingly popular and widespread (Pirani & Vignoli, 2016; Pirani et al., 2021). Meanwhile, from a socioeconomic perspective, the role of women has notably shifted, reflected in higher educational attainment and greater labour market participation. However, societal arrangements and welfare provisions, such as gender role equality or flexible working conditions favouring work-life balance, have not advanced at the same pace. In Italy, a marked process of work precarisation has furthermore occurred. Together with the recent economic downturns, this has strongly deteriorated labour market positions, especially for young adults (Lin et al., 2013; OECD, 2012; Pirani & Salvini, 2015).

In this rapidly changing context, evidence of the relationship between union formation and economic conditions is scarce, and relevant contributions are based on information that is now outdated (Bernardi et al., 2005; Kalmijin, 2011; Vignoli et al., 2016). Here, we employ the most recent available data—the ‘Families and Social Subjects’ survey conducted by the Italian National Institute of Statistics (ISTAT) in 2016﻿—and consider men and women aged 25–44 years at the time of the interview, following them retrospectively from 1995 to 2015, namely from the age of 16 until union formation or survey date. Given that men and women differ in patterns of entry into co-residential unions (Bolano & Vignoli, 2021; Vignoli et al., 2016; Wiik, 2009), we conduct our empirical analyses separately by gender. Similarly, in the light of persistent differences between marriage and cohabitation in the Italian context (see, e.g. Pirani & Vignoli, 2016), we also account for union type.

The paper is organised as follows. Section 2 provides a background description and presents our hypotheses in the Italian context. Section 3 describes the data and analytical strategy, followed by a discussion of our results in Sect. 4. In the final section, we offer concluding reflections on our findings.

## Background

### Micro-Level Economic Vulnerability and Union Formation

Though the decision to start a co-residential union is primarily emotional (Oppenheimer, 1988), it also involves investments of time, money and psychological resources. A source of income is a prerequisite for starting a co-residential union (Kalmijn, 2011) and having a job is the most common means to this end. Financial security should, however, be considered prospectively; the mere existence of an income may not be sufficient. A critical factor could be a sense of security about future economic prospects, making non-standard jobs or temporary employment inadequate preconditions for starting a co-residential union. In the presence of financial instability, postponing a co-residential union could be a strategy to avoid risk, as postulated by the *uncertainty hypothesis* (Oppenheimer, 1988). With these considerations in mind, scholars (e.g. Kalmijn, 2011; Schneider et al., 2019; Vignoli et al., 2016) have highlighted the importance of accounting for aspects other than being employed or not in defining individuals’ economic vulnerability.

In contrast, according to the *uncertainty reduction* theory (Friedman et al., 1994), entering a union may represent a strategy to reduce biographical uncertainty and respond to negative employment prospects, particularly for women. Especially in societies where men are primarily responsible for family income—male-breadwinner societies—men’s economic situation might be more important than women’s for union formation (Raz-Yurovich, 2010). In this view, unemployed men or those in unstable employment are considered less attractive as partners, which may reduce their likelihood of forming a co-residential union. In contrast, women with poor employment prospects may choose to form a union as a strategy to reduce uncertainty. Conversely, women who are no longer dependent on men’s economic conditions may be less likely to form a union, and, at the same time, those with more prosperous economic prospects may be less attractive for co-residential union formation (Becker’s (1991) *women’s economic independence* hypothesis).

These frameworks are especially applicable in societies with a high degree of specialisation in gender roles (Bernardi et al., 2005; Ongaro, 2001). The argument is that the importance of a woman’s economic condition for union formation depends on the degree of gender equality in society (Thomson & Bernhardt, 2010). In contexts shifting from a male-breadwinner to a dual-breadwinner model, a stable employment situation for both the man and the woman becomes a prerequisite for family formation (Kreyenfeld et al., 2012). Likewise, the economic vulnerability of women, besides that of men, represents an essential factor in postponing co-residential unions. Empirical studies using USA, French and Korean data show that the differences between the roles of male and female economic situations have attenuated in recent cohorts (Kim, 2017; Schneider & Reich, 2014; Schneider et al., 2019; Vergauwen et al., 2016).

Moreover, it has been documented that the type of co-residential union matters, with the impact of economic uncertainty arguably differing between marital and non-marital cohabitation. Indeed, given different normative expectations regarding these two ways of forming a union, employment status, employment characteristics and individuals’ more general economic circumstances may be less critical for unmarried cohabitation than for marriage (Bukodi, 2012; Kalmijin, 2011; Jalovaara, 2012; Xie et al., 2003). Various scholars suggest that cohabitation is more compatible with individual economic vulnerability (Mills & Blossfeld, 2013; Oppenheimer, 2003; Perelli-Harris et al., 2010; Schneider et al., 2019; Sassler & Lichter, 2020) due to its temporary and reversible nature, compared to the stronger normative expectations of marriage as a long-term commitment. The underlying idea is that until individuals reach a certain level of job stability, secure enough to support a future family economically, they may prefer to postpone marriage and opt for a less formal union, such as cohabitation. Various studies have, in fact, documented that individuals in more uncertain economic positions are more likely to enter cohabitation (Bukodi, 2012; Guetto et al., 2020; Vignoli et al., 2016).

### Macro-Level Economic Conditions and Union Formation

Macro-level economic conditions can influence union formation in two ways. First, a stagnant and poor economic and financial context implies unfavourable employment prospects, like scarce job offers, high unemployment and poor and temporary working conditions. Globalisation has, for instance, led to an increase of (young) people entering the labour market with temporary or casual contracts (Blossfeld & Mills, 2005; Mills & Blossfeld, 2003). Similarly, the recent economic recession has raised the individual likelihood of unemployment, job instability, and insecurity. From this perspective, the relationship between macroeconomic adversity and union formation—and, more generally, individuals’ life courses—could be (at least partially) explained by individuals’ employment situations as a compositional effect.

Second, the economic context may directly affect decisions by strengthening or weakening individual-level economic insecurity and thus reducing or enhancing the likelihood of starting an independent life with a partner. That is, under prosperous macroeconomic conditions, even unemployed or temporarily employed individuals might be optimistic about their labour market (and economic) circumstances in the near future, despite their current economic vulnerability. Similarly, during an economic downturn, even individuals with permanent employment might feel pessimistic about future labour market prospects (e.g., fears of job loss, reduced chances of promotion, and wages less likely to be adjusted to inflation) and delay union formation decisions. Understanding how the macroeconomic context acts as an autonomous contextual effect may help shed light on the link between economic conditions and the union formation process.

Although a substantial body of research reveals the importance of aggregate-level economic aspects for individual family behaviours (relative to fertility patterns see, for instance, Aassve et al., 2006; Neels et al., 2013), less attention has been paid to whether both micro- and macro-level factors impact union formation. To our knowledge, this question has only been addressed by two country-specific studies. De Lange et al. (2014) empirically demonstrate that, in the Netherlands, an unfavourable macroeconomic situation delays the first co-residential union, and the negative effect of macroeconomic adversity persists when controlling for individual economic conditions. Thus, an adverse economic context increases a sense of uncertainty regarding future employment and economic prospects (Vignoli et al., 2020), resulting in the postponement of union formation, even for those with more favourable (namely stable) employment positions. Vergauwen et al. (2016) observe a similar dynamic for France, also documenting differences in the effect of the macro-level economic situation according to gender. Specifically, they suggest that women might be less affected by the economic context due to both their reduced labour market prospects compared to men and the fact that they are more frequently employed in the service and public sectors, less sensitive to changes in economic conditions. No study has explicitly considered—net of micro effects—the potentially different impact of macroeconomic uncertainty on marriage versus cohabitation.

### The Italian Context

Italy has experienced profound economic, demographic and cultural changes in recent decades, making it an interesting context to explore whether and how both macro- and micro-economic conditions impact the union formation process.

In recent decades, unprecedented levels of uncertainty have been seen in labour markets across the Western world, and Italy is no exception. Labour market flexibilisation began in the 1990s and, due to several subsequent reforms, continued for a number of years (see Fana et al., 2015 for a detailed description). An explosion of flexible and temporary contract forms resulted, characterised by lower wages and limited social protection (Tompson, 2009). This shift occurred remarkably quickly—one of the fastest in Europe (OECD, 2016)—and significantly affected Italian workers (Barbieri & Scherer, 2009; Fana et al., 2015). While temporary work opportunities may have partly contributed to a slight decrease in the youth unemployment rate at the beginning of the 2000s, generally, these changes negatively impacted workers’ employment biographies, decreasing the possibility of obtaining stable long-term employment and reducing career prospects (Barbieri & Scherer, 2009). The economic crises of the new century exacerbated this situation. Italy was among the countries hardest hit by the recent economic downturn beginning in 2007, the so-called Great Recession, partly due to the country’s labour market and institutional characteristics. Employment rates dropped and workers experienced substantial job losses, especially those with temporary contracts (Lin et al., 2013; OECD, 2012). The consequences were particularly severe for youth, the low and middle socioeconomic classes, and migrants (Brandolini et al., 2018).

Italy further presents an intriguing setting, given its particular demographic and cultural characteristics. Long a conservative society in terms of family dynamics, the country has recently begun transitioning to less traditional family and gender behaviours. Though Italians have typically viewed marriage as a fundamental step in the transition to adulthood (Billari & Rosina, 2004; Ongaro, 2001), in the 1990s the centrality of marriage in family life began to waver (Pirani & Vignoli, 2022; Pirani et al., 2021), and non-marital cohabitations have gradually gained acceptance as a socially viable form of union and possible alternative to marriage. Among those who left their family of origin to form a union before the age of 30, less than 2% of those born in 1950–55 chose a non-marital cohabitation. This figure rose to around 20% for those born in 1970–74 and further increased for subsequent cohorts (ISTAT, 2014). Although an apparent territorial heterogeneity persists across Italian regions (e.g. Aassve et al., 2024), there has been a notable shift towards complete acceptance of cohabitation in the new millennium. Italy is also interesting from a gender perspective. Despite an ongoing social and cultural change towards greater gender equality—for instance, in education (ISTAT, 2021; World Economic Forum, 2022)—the country is still characterised by marked inequalities in both the labour market and family life (e.g., Altintas & Sullivan, 2016; Dotti Sani, 2018). Women’s labour market participation is relatively low compared to other European countries, and more women than men are employed in jobs characterised by higher precariousness and poorer conditions (Pirani & Salvini, 2015). This scenario could suggest that the male-breadwinner model may not completely disappear in Italian society (Anxo et al., 2011; Menniti et al., 2015). This changing demographic and societal Italian context makes understanding the impact of both microeconomic vulnerability and macroeconomic uncertainty on union formation paramount.

### Research Hypotheses

In the light of the theoretical framework and particularities of the Italian context, we formulate two sets of hypotheses for the relationship between union formation and micro- and macro-level economic conditions.

HP 1. In line with the uncertainty hypothesis, individual economic vulnerability due to unfavourable labour market positions is posited to reduce the likelihood of first union formation. Nevertheless, differentiated effects by union type and gender are expected.

HP 1a. We foresee that individual economic vulnerability reduces the likelihood of marrying to a greater extent than that of cohabiting, based on the different commitment and normative value of marriage versus non-marital cohabitation (see, for instance, Oppenheimer, 2003), especially in a country of late cohabitation diffusion like Italy. We thus deem that cohabiting unions are more compatible with insecure employment statuses than marriage, at least in the first phase of family formation.

HP 1b. The role of individual economic vulnerability is posited to be stronger for men than for women, especially in the case of transition to marriage. Due to the traditional gender norms that still characterise Italian society along with the related potential endurance of the male-breadwinner model, we expect that women in economically vulnerable circumstances are less prone to delay a union than men. From this perspective, according to the uncertainty reduction theory (Friedman et al., 1994), women may reduce their biographical uncertainty by investing in a family career instead of a working one. This strategy, however, may be hypothesised to be particularly effective for marriage rather than for cohabitation, given the greater legal protections afforded to married women.

HP 2. Net of individual labour market position, economic uncertainty at contextual level is suggested to reduce the likelihood of first union directly. Differences by union type and gender are again hypothesised in this case.

HP 2a. Given the different meanings of marriage versus non-marital cohabitation, we assume that concerns about an unstable macroeconomic environment are more likely to influence young adults to initiate a less reversible and long-term commitment union, such as marriage, rather than cohabitation.

HP 2b: Due to the possible residual persistence of the male-breadwinner model in Italian society, we expect that women are less susceptible than men to the potential future consequences of adverse macro-level economic conditions on their occupational status.

## Empirical Investigation

### Data and Methods

We rely on retrospective data from the ‘Families and Social Subjects’ survey conducted in Italy by the Italian National Institute of Statistics (ISTAT) in 2016 on a representative sample of 24,753 people aged 18 years and over. This survey represents the most complete, up-to-date and reliable source for Italy, encompassing a broad range of demographic, social and economic characteristics of individuals and their families, including detailed information on individuals’ partnership and employment histories (e.g. type of contract of each job).

Our analytical sample comprises men and women aged 25–44 years at the time of the survey, thus born between 1972 and 1991. These individuals are followed retrospectively from the age of 16 years until the first union formation or the survey date, whichever comes first. Among the 7,122 individuals in our analytical sample—3,503 men and 3,619 women—two-thirds had started their first co-residential union before the interview (60% of men and 72% of women). Direct marriage was slightly more prevalent among women (58% of first unions were a direct marriage) than among men (50% of first unions were a direct marriage).

Following the standard approach in the literature, we study the correlates of entry into marriage or cohabitation through the discrete-time event history model in a competing risks approach, which in practice entails the estimation of a multinomial logistic regression model (e.g. Berrington et al., 2000; Graaf and Kalmijin, 2003; Vignoli et al., 2016). We are aware that the presence of unmeasured factors that simultaneously influence the entry into marriage or cohabitation may lead to the violation of the independence of irrelevant alternatives (e.g., Hill et al., 1993; Kalmjin, 2011), thus entailing biased estimates. Nevertheless, the preliminary estimation of a multilevel discrete-time event history model with correlated random effects to allow for shared unobserved risk factors (Haan & Uhlendorff, 2006; Steele et al., 2005) proved the non-significance of the correlation between the error terms in our case (results not shown here but available upon request). Therefore, we ultimately opted for the standard multinomial logistic approach to follow a less computationally intensive approach and ensure comparability with previous findings (e.g. Vignoli et al., 2016). We thus created a person-years dataset, tracking respondents annually from the age of 16 (the start of the process)^1^ until the event of interest (marriage or cohabitation) occurred. Respondents who had not entered their first union before the interview were censored at the time of the survey. We also estimated separate models for men and women.

### Correlates of Union Formation

#### Approximating Micro- and Macroeconomic Conditions

Our focus is on economic conditions, both at the micro- and macro-levels. At the individual level, due to data availability constraints, we proxy economic vulnerability using the respondent’s employment status, namely permanently employed (the lowest degree of personal economic vulnerability); self-employed (a heterogeneous category, possibly entailing various levels of uncertainty^2^); temporarily employed (including fixed-term contracts and so-called casual workers^3^); and not employed^4^ (the highest degree of personal economic vulnerability). Employment histories, including information on the type of contract for each job, are recorded retrospectively on a monthly basis, allowing us to consider this categorical variable as a time-varying covariate.

For macro-level economic uncertainty, among the possible measures suggested in the previous literature (e.g., De Lange et al., 2014; Lappegård et al., 2018; Sobotka et al., 2011), two indicators are used in the current paper. First, we opted for the annual time series of the unemployment rate (ages 15–24) drawn from ISTAT. Unemployment rates have frequently been used as indicators of the economic context in the literature on union and family formation (e.g. Sobotka et al., 2011), representing a measure that could directly affect individuals’ lives due to its potential link to lack of employment (or insecure prospects) at the individual level. When considering the most appropriate territorial level, the national level should be discarded as too general, especially in a heterogeneous country like Italy, where territorial mobility is limited. We thus decided to exploit the most disaggregated level allowed by the data—the regional level or NUTS level 2 area. We also tested other measures that broadly reflect the state of the labour market—e.g. activity rate, occupation rate and unemployment rate for different age ranges—none of which substantially varied the results. Second, we introduced the current annual consumer confidence index (CCCI), which was again provided by ISTAT. This indicator, which has been shown to capture the effect of the economic context on family behaviours more effectively than other measures, such as GDP or inflation rate (Sobotka et al., 2011; Vergauwen et al., 2016), is designed^5^ to assess the optimism/pessimism of consumers, thus providing a complete picture of the climate of the country beyond economic and labour market conditions. CCCI is intended as a general indicator of economic conditions, a sort of subjective perception (at the collective level) of present and future economic prospects. The preliminary analysis found no territorial variations for the Italian macro-areas (NUTS level 1). Therefore, we opted to include this index at the national level, with a primary focus on temporal variations.

For the sake of comparability and ease of interpretation, in our model specification, we introduced the two measures of macro-level economic uncertainty in a standardised version, so that they range between 0 and 1 (min–max normalisation). Moreover, both these macro-level indicators were lagged by 1 year.^6^

**Fig. 1:**
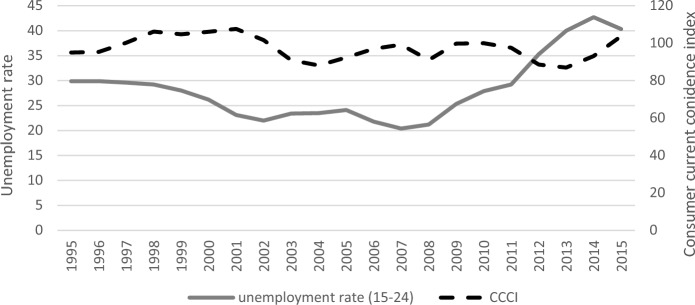
– Youth unemployment rate (left axis) and current consumer confidence index (CCCI, right axis). Years 1995–2015. *Source:* ISTAT data

Figure 1 displays the trend of the two macro-level variables for the period under consideration at the country level (and in the original unit of measurement). The youth unemployment rate (left axis) was around 30% in the mid-’90 s, followed by a slow, progressive decrease in connection with the labour market reforms of that period. Specifically, this decline of 6–10 percentage points was primarily due to the introduction of more flexible and temporary forms of work contracts, which alleviated youth unemployment. However, beginning in 2007 with the start of the Great Recession, the youth unemployment rate (as well as the total unemployment rate) increased sharply year after year, surpassing 40% in 2013.

The CCCI (right axis) displays a more irregular trend (2010 = 100). The index values progressively increased through the late’90 s and into the early 2000s (from 95 to 108), followed by a drop, a slight recovery, and then a new negative peak (91 in 2008) in the first decade of the new millennium. There has been a rising trend in the CCCI in recent years, reaching 105 in 2015.

We acknowledge the possibility of some overlap between the unemployment rate and the CCCI, both being related to the economic context. Indeed, we found a certain correlation (−0.43, significant at 10% level) between them, when the unemployment rate was lagged behind. We ran robustness checks considering the indicators one at a time in the model specification, achieving virtually identical results as when considering the two measures jointly. This convinced us that, despite any weak correlation that may exist, the two measures can capture two different facets of the economic context.

#### Micro-Level and Macro-Level Confounding Variables

Regarding micro-level control variables possibly associated with the union formation process, we accounted for both individual and family characteristics. Among the former, we considered several individual-level characteristics shown in the literature to be relevant factors (Harknett & Kuperberg, 2011; Jalovaara, 2012). Where not explicitly stated, all the following characteristics have been constructed in their time-varying specification. The individual’s age, categorised into five classes (16–20, 21–24, 25–29, 30–34 and 35 and up), represents the baseline duration, or the time passed since the age of 16 years. Educational level distinguishes between lower-secondary, upper-secondary and higher education, and is accompanied by a dichotomous covariate that defines student enrolment. We also considered whether the respondent has children and if they left the parental home for non-union-related reasons (Meggiolaro & Ongaro, 2024). For family background, we took into account parental socioeconomic status (SES) and parental marital status. Indeed, high parental SES has been found to delay the timing of the first union, especially in the case of marriage (e.g., Brons et al., 2017; Mooyaart, 2019; Mooyaart & Liefbroer, 2016; Wiik, 2009). Meanwhile, young adults with separated parents are more likely to enter their first union earlier—especially a cohabiting union—than those with intact families (e.g,. Härkönen et al., 2021; Mazzuco & Ongaro, 2009; Perelli-Harris et al., 2017). Specifically, we used the mother’s level of education (secondary or lower, and tertiary) and her occupational status (employed or not)—in this case, both measured when the respondent was 15 years old—to proxy parental SES, and included a time-varying indicator of parental separation. Although not directly linked to our research questions, these variables may contribute to deciding whether to enter a first union and decide to marry versus cohabit.

Table 2 in the Appendix presents the distribution of exposures (person-years) and occurrences of marriage and cohabitation by socio-demographic characteristics and sex. Among other things, we observe that the average age at first union is relatively high, especially in the case of marriage (though lower for women than for men). Socio-demographic and parental characteristics reflect previous evidence: cohabiting unions are more diffused among highly educated youth (especially women) from families with mothers who are highly educated and who participated in the labour market. As for our individual-level key covariate, cohabitations are somewhat more common than marriages among young men with temporary work. Non-working women are overrepresented among those who are married (this group includes inactive women—that is, housewives). Finally, non-marital cohabitations become more frequent, especially in recent years, and a North–South gradient is evident.

We used two covariates as controls for the macro-level context: calendar time (1996–1999, 2000–2003, 2004–2007, 2008–2011, 2012–2015) and area of residence (North, Centre and South/Isles). Both provide proxies of the cultural, structural and economic context and, in the complete model specification including all micro- and macro-level economic variables, should capture the residual (cultural and structural) aggregate effect on the union formation process.

## Results

In this section, we present the results from the discrete-time competing risks models, comparing the risk of entering first marriage or first cohabitation separately for men and women. Table 1 shows, for the model specifications described below, the results in terms of relative risk ratios (RRR) of the variables approximating the micro- and macroeconomic conditions (complete results of the three model specifications are reported in Tables 3 and 4 in the Appendix). Model 1 includes the micro-level economic condition, controlling for all socio-demographic confounding variables at the individual level. This model allows us to assess the impact of microeconomic vulnerability on the propensity to marry and cohabit, without controlling for the (possible) effects of macro-level factors. Model 2 adds calendar time and area of residence, two macro-level variables that should account for the changing (cultural, economic and institutional) context by time and geographical area. This model specification is intended to verify HP 1, namely, if and to what extent first union formation correlates with the individual’s employment situation, controlling for a possible effect of the context. Finally, Model 3 adds the two key macro-level economic covariates (unemployment rate and CCCI), allowing us to verify the existence of a direct effect of macro-level adverse economic conditions on the transition to marriage and cohabitation, all else equal. This enables us to verify HP 2.

**Table 1:** Results from discrete-time competing risks models of the transition into first marriage and first cohabitation: relative risk ratios (RRR). Separate models by sex. *Source*: Authors elaborations of FSS Italian data. Models controlled for micro- and macro-level confounding variables: age classes, educational level, student status, having had children, having left parental home for non-union-related reasons, parental separation, mother’s level of education and occupational status, calendar time, area of residence (see Table 3 and 4 in the Appendix for complete models results)

	MEN						WOMEN					
	Mod. 1		Mod. 2		Mod. 3		Mod. 1		Mod. 2		Mod. 3	
	RRR	*sig.*	RRR	*sig.*	RRR	*sig.*	RRR	*sig.*	RRR	*sig.*	*RRR*	*sig.*
**Marriage**												
*Individual employment (ref.: permanent)*												
Self-employment	1.016		0.950		0.949		1.023		0.975		*0.976*	
Temporary employment	0.706	***	0.667	***	0.667	***	0.765	**	0.747	**	*0.747*	**
No work	0.360	***	0.315	***	0.314	***	0.742	***	0.649	***	*0.651*	***
*Unemployment rate (15-24)*					1.121						*0.872*	
*CCCI*					0.938						*1.356*	
**Cohabitation**												
*Individual employment (ref.: permanent)*												
Self-employment	0.842	*	0.936		0.941		0.726	**	0.771	*	*0.779*	*
Temporary employment	0.813	**	0.896		0.904		0.848	*	0.900		*0.902*	
No work	0.332	***	0.426	***	0.432	***	0.350	***	0.429	***	*0.433*	***
*Unemployment rate (15-24)*					0.381	**					*0.578*	*
*CCCI*					0.774						*1.213*	

*** = *p* < 0.001; ** = *p* < 0.05; * = *p* < 0.10

### Transition into Marriage

In Model 1, we see that compared to those permanently employed, temporarily employed individuals have a reduced risk of entering into marriage (RRR equals 0.706 for men and 0.765 for women). The negative effect is even stronger for those who don’t have a job, especially men (RRR = 0.360). However, it is not negligible also for women (RRR = 0.742), in contrast with the idea of a male-breadwinner society and previous findings for Italy. Self-employed men and women are not found to be significantly different from their permanently employed counterparts.

These individual-level effects persist and even enlarge in Model 2, which controls for calendar time and area of residence. Accounting for the (cultural, institutional or economic) context and having temporary compared to permanent employment significantly reduces the likelihood of marriage (RRR equals 0.667 for men and 0.747 for women). The negative effect is even more marked for individuals out of the labour market (RRR = 0.315 for men; RRR = 0.649 for women). These findings suggest that not only men but also women have a reduced risk of entering into marriage if they experience economic vulnerability. Interestingly, in general terms of period and area of residence, the context significantly affects the risk of entering into marriage for both men and women (see also Tables 3 and 4 in the Appendix and Sect. 4.4). However, this model specification does not allow us to isolate the effects of the different environmental factors, leading to Model 3.

The two macroeconomic covariates included in Model 3 show that—net of individual occupational position—economic uncertainty at the contextual level has a specific effect, especially for women (with a weak statistical significance): higher consumer confidence levels increase the likelihood of marrying (RRR = 1.356). A more favourable economic climate thus increases the likelihood of first marriage for women but not for men, an unexpected result.

### Transition into Cohabitation

As for the transition into the first cohabitation, Model 1 shows that micro-level vulnerability has a negative impact, regardless of gender. Compared to permanent employment, temporary employment decreases the likelihood of entering into cohabitation (RRR = 0.813 for men; RRR = 0.848 for women). The same applies to non-working respondents, with a larger magnitude (RRR = 0.332). Additionally, self-employed men have a moderately lower probability of entering into cohabitation, an effect that is probably due to the heterogeneous composition of this category: whereas entrepreneurs and professionals can be assimilated to permanent workers, independent workers and freelancers generally work on a more casual basis, thus entailing a higher degree of job (and economic) uncertainty.

When controlling for calendar year and area of residence (Model 2), the association between temporary employment and union formation disappears. A negative effect is still found, although with a slight reduction in the magnitude, only for individuals out of the labour market. Compared to the permanently employed, both men and women without a job show a decreasing propensity to enter into a first cohabitation (RRR = 0.426 for men; RRR = 0.429 for women). A moderate effect is also found for self-employed women. It would thus seem that an unstable position in the labour market is compatible with cohabitation, perhaps due to the lower level of commitment characterising this type of union and its more easily reversible nature than marriage.

In Model 3, we see that, net of individual position in the labour force, when macroeconomic conditions deteriorate, individuals are less likely to enter into cohabitation. This is especially true for men, for whom the unemployment rate is significantly and negatively associated with first cohabitation (RRR = 0.381). For women, the effect is weaker and only slightly significant (RRR = 0.578). These findings suggest that—unlike with marriage—macro-level economic uncertainty may reduce the likelihood of starting a first cohabitation, for both men and women.

### In Summary

Overall, our hypotheses were only partially confirmed. Our findings suggest that economic vulnerability, both at the individual and macro level, reduces the likelihood of forming a union, confirming HP 1 and HP 2. This association, however, has been found to vary by gender and union type, and not always in the direction of our expectations.

We predicted that entry into marriage would be more negatively affected by individual economic vulnerability than entry into cohabitation (HP 1a). Our findings confirmed this hypothesis: Not having a job reduces the probability of forming the first union regardless of the type of union while having a temporary job reduces the likelihood of marrying but does not matter for cohabiting.

We also predicted that the role of individual economic vulnerability would be stronger for men than for women (HP 1b), but we found no evidence supporting this hypothesis. Indeed, no gender difference emerged for those temporarily employed or those not employed. This last result may be due to the impracticality of distinguishing unemployment from inactivity in the available data. The broad category of not employed women could include a non-negligible percentage of unemployed women, who may be less prone to invest in family (and marriage) than inactive ones. Weak evidence of a negative effect on entering into cohabitation is found for self-employment, but only for women.

As for macro-level economic vulnerability, HP 2a posited that an unstable economic context at the macro level is likely to influence marriage more than cohabitation. Contrary to our expectations, we found it more relevant for entering cohabitation than marriage. Once more, differences between men and women are not straightforward and are intertwined with union type. While women appear to be less affected than men by macro-level uncertainty when it comes to entry into cohabitation (in line with HP 2b), no evidence was found for the impact of this factor on their entry into marriage.

To strengthen the interpretation of this result, we estimated a series of additional specifications, explicitly addressing a potential overlap between the macro-level economic indicators and the calendar time (Models 4–6 reported in the Appendix, Tables 5 and 6). It is straightforward that dropping the calendar time from our last model specification (Model 4) expands the statistical effect of the macro-level economic indicators. However, if we introduce two other macro-level indicators intended to capture variations in the *cultural* context possibly linked to union formation—cohabitation diffusion and secularisation^7^—these effects again scale down (Model 5). Indeed, the turn of the new century has seen both a significant increase in unemployment and exceptional diffusion of family-related behaviours in the Italian context (e.g. Pirani & Vignoli, 2016; Aassve et al., 2024), and the absence of a contextual control could lead to misleading (overestimated, as in Model 4) results. Model 5 yielded results congruent with those of Model 3, and given the objectives of our study, we preferred the specification including the calendar period as a general control—this choice aimed to capture residual effects at the contextual level that may have occurred over time. Model 6 further confirmed these results. Interestingly, Model 5 helps demonstrate that the *cultural* explanation is more potent than the economic one in predicting the likelihood of entry into marriage.

### Control Covariates

The outcomes for the individual control variables are generally unsurprising and align with the literature (see Tables 3 and 4 in the Appendix, here we refer to the complete Model 3 specification).

Compared to those aged 25–29 years, younger individuals have a lower risk of entering a first union, which holds for marriage and cohabitation (along with women aged 35 years and older, who have the lowest risk of cohabiting). In addition, student status reduces union formation probability (except for cohabitation among men). In terms of education, people with upper-secondary education levels have a lower risk of entering a union than those with higher or lower levels of education, except for women with high levels of education, who have a lower risk of cohabiting relative to those with low education levels. As expected, parenthood increases the risk of entering a union (both marriage and cohabitation), regardless of gender.

Several other socio-demographic individual covariates might shape the decision to marry or cohabit as the first form of union. Having left the parental home for reasons other than union formation decreases the risk of entering into marriage for women (but not for men) and increases the risk of entering into cohabitation for both. This is an interesting result that has not often been considered in the literature, and in a certain sense, enables us to account for a *piece* of individuals’ life trajectory (see also Meggiolaro & Ongaro, 2024). Meanwhile, in terms of family background, women with separated parents are less likely to marry and more likely to enter cohabitation. Men with higher-educated parents are less likely to marry, and men and women whose parents have upper-secondary education levels have a higher risk of entering into cohabitation.

Finally, macro-level control variables, such as period and area of residence, play a role, though their effect weakens after including macro-level economic variables. Young people living in Southern Italy have a higher risk of entering into marriage and a lower risk of entering into cohabitation than those in the Northern or Central regions. Similarly, the effect of calendar time remains significantly negative for marriage in more recent years (for both men and women). In contrast, that for cohabitation (only for women) shows a clear negative trend before 2000 compared to the subsequent periods. These findings indicate a residual impact of the context (time and place) on the union formation process, which depends on (presumably cultural) differences associated with the time periods (see, e.g., Model 6) and geographical areas considered by the study (see Sect. 2.3).

## Discussion and Conclusion

This study examines the role played by individual labour market positions (measured in terms of employment status) on the probability of forming the first co-residential union and additionally explores whether adverse macroeconomic conditions contribute to this dynamic. In investigating this question, we differentiate by union type (marriage and cohabitation) and gender.

We focus on Italy, a fascinating setting given the many demographic, socioeconomic, and cultural changes the country has experienced in the last few decades. Since previous research on this topic in Italy is based on outdated information, more recent data allows a better examination of the relationship between economic factors and union formation in a changing context.

We find that while both micro- and macro-level economic factors matter in the union formation process, their effects crucially depend on gender and union type. To the best of our knowledge, we are among the first to investigate the extent to which both micro- and macroeconomic circumstances account for these distinctions, thus demonstrating their importance. This evidence is relevant for a country like Italy, where gender equality remains far from being realised, and differences between marriage and cohabitation persist.

We document that individual economic vulnerability has a greater impact on marriage than on cohabitation, though the same is not proven for macro-level economic conditions. Contrary to De Lange et al. (2014), who find that individual employment insecurity does not contribute to postponing marriage in the Netherlands, we show that when considering entry into marriage, an individual’s employment status is more relevant than macroeconomic conditions, with the latter having a weak effect only for women. In contrast, macroeconomic conditions do have an impact on entry into cohabitation for both men and women. One possible explanation is that—at least for a country like Italy—the decision to take the important step of marrying is mainly driven by private and personal motivations and less influenced by the external economic context. In other words, in a cultural context where marriage is still highly valued and seen as a significant milestone in adult life, individual economic circumstances may play a more prominent role in the decision-making process, with less emphasis given to external factors. In contrast, while more easily combined with a precarious economic position at the individual level (e.g. temporary contracts), non-marital cohabitation might be hindered by uncertain contextual conditions. Because of the different investments entailed by the two union types—greater for marriage and lower for cohabitation—the former is primarily influenced by individual uncertainties rather than contextual uncertainties. We also incidentally found that the cultural explanation is more potent than the economic one for the probability of marriage.

Another novel result relative to previous literature (and contrary to our expectations) is that women are not less influenced by micro- and macroeconomic vulnerability and uncertainty than men, as one might presume based on the male-breadwinner family model. This finding contrasts with the work of Vergauwen and colleagues (2016), who document that, in France, macro-level conditions are linked to union formation only for men and that inactive women are the most likely to form a partnership, suggesting that the male-breadwinner model has not disappeared. Differences between our and their findings can be explained by the diverse period of observation of the two studies—the current one enlarging the observation window till 2015—and the diverse specification of the “no work” category. However, it can be argued that although Italy maintains significant traditionalism in gender roles and family behaviours, these dynamics are likely changing among the younger generations. The steady increase in (female) educational levels and the progressive diffusion of more egalitarian gender roles likely contribute to the erosion of the male-breadwinner model. This may also be connected to the fact that in the first decade of the 2000s, average wages in Italy began to decline relative to the average among OECD countries (OECD, 2024), and two sources of income are a requisite to enter cohabitation. In this context, the current employment situation and future prospects of the female partner have also started to emerge as crucial factors in the union formation process, which aligns with the different roles of men’s and women’s labour market positions identified by Vignoli and colleagues (2016) in their examination of earlier data.

Several implications of our findings are worth considering. Delayed achievement of economic autonomy may hinder the transition to adulthood for the younger Italian generation. Having (secure) employment is a prerequisite for forming a union for both partners, not just for the man, and the two effects together could imply a further postponement of union formation. This step may compromise union formation and subsequent related life course events if this happens relatively late. The increasing diffusion of cohabiting unions, which we find to be less susceptible to individual economic uncertainty than marriages, should not, however, be considered a strategy to overcome the adverse effect of economic uncertainty. Indeed, more so than marriages, cohabitations are influenced by macro-level economic conditions. In this context, policies might focus on ways to facilitate the achievement of young adult economic autonomy.

Despite the strengths of the papers, we acknowledge some limitations in our work. In measuring individual-level economic vulnerability, first, we missed information on individuals’ economic and financial conditions or other contract characteristics, which could help identify unusual uncertainty situations. Second, we were not able to differentiate non-working people between unemployed and inactive. This can be challenging, especially for women, for whom the distinction between unemployment and housekeeping is relevant. As for the macro-level source of uncertainty, while this study is among the few that account for it, we recognise the need for more specific details, potentially at a more granular territorial level. This would enable a more accurate depiction of the environmental conditions in which individuals are embedded. In addition, while focusing on the first union is interesting from the point of view of social and normative dimensions of the family formation process, data constraints prevented us from adopting a couple perspective, considering, for instance, information on a partner’s job position or economic status (van Wijk et al., 2021). Future research employing more detailed data might investigate these aspects. Finally, it is worthwhile to remember that non-marital cohabitations, usually referring to a living arrangement between two intimate partners who are not married, can be adopted for many reasons, thus entailing several characteristics, values and levels of commitment. People choose to cohabit as an alternative to marriage, but also as a step in a couple’s life that will eventually lead to marriage, or simply as an alternative to living alone. Recent decades have shown a dramatic increase in the diffusion of cohabitation in Italy (e.g. Pirani & Vignoli, 2016). Whether it is undeniable that in the traditional Italian setting, the pioneers of the diffusion process have been highly educated, liberal and open-minded people, in line with the Second Demographic Transition narrative, it is also true that the educational gradient is currently vanishing (e.g. Aassve et al., 2024), with an increasing spread of cohabiting unions across all social groups, included the more economically disadvantaged. All of this raises the question of whether different paths leading to cohabitation are influenced differently by individual and contextual (economic) factors. We deem that different motivations underlying the cohabiting decision need a fresh look, thus soliciting social scientists and data providers also to consider these aspects to gain a more comprehensive understanding of a phenomenon that has become increasingly widespread, even in the Italian context.

## Data Availability

The data that support the findings of this study are available from ISTAT but restrictions apply to the availability of these data, which were used under licence for the current study, and so are not publicly available.

## Appendix

See Tables 2, 3, 4, 5 and 6.

**Table 2:** Exposures (person-years) and occurrences of marriage and cohabitation, by socio-demographic characteristics and sex: Absolute and percentage values

	MEN						WOMEN					
	Exposures		Marriage		Cohabitation		Exposures		Marriage		Cohabitation	
	abs. val.	*%*	abs. val	*%*	abs. val	*%*	abs. val	*%*	abs. val	*%*	abs. val	*%*
	39,256	*100.0*	995	*100.0*	1,005	*100.0*	32,878	*100.0*	1,343	*100.0*	1,004	*100.0*
*Individual's age*												
16-20	10,840	*27.6*	22	*2.2*	97	*9.7*	10,652	*32.4*	118	*8.8*	142	*14.1*
21-24	11,185	*28.5*	144	*14.5*	216	*21.5*	9,936	*30.2*	361	*26.9*	272	*27.1*
25-29	10,352	*26.4*	443	*44.5*	414	*41.2*	7,752	*23.6*	604	*45.0*	385	*38.3*
30-34	4,650	*11.8*	304	*30.6*	210	*20.9*	3,028	*9.2*	210	*15.6*	165	*16.4*
35+	2,229	*5.7*	82	*8.2*	68	*6.8*	1,510	*4.6*	50	*3.7*	40	*4.0*
*Employment*												
Permanent	12,888	*32.8*	535	*53.8*	527	*52.4*	8,270	*25.2*	496	*36.9*	464	*46.2*
Self-employment	4,471	*11.4*	198	*19.9*	155	*15.4*	1,579	*4.8*	98	*7.3*	71	*7.1*
Temporary	5,235	*13.3*	120	*12.1*	165	*16.4*	4,256	*12.9*	170	*12.7*	197	*19.6*
No work	16,523	*42.1*	140	*14.1*	149	*14.8*	18,652	*56.7*	574	*42.7*	270	*26.9*
n.a.	139	*0.4*	2	*0.2*	9	*0.9*	121	*0.4*	5	*0.4*	2	*0.2*
*Parental separation *												
No	36,010	*91.7*	933	*93.8*	893	*88.9*	29,912	*91.0*	1,269	*94.5*	843	*84.0*
Yes	2,298	*5.9*	35	*3.5*	91	*9.1*	2,006	*6.1*	47	*3.5*	130	*12.9*
n.a.	948	*2.4*	27	*2.7*	21	*2.1*	960	*2.9*	27	*2.0*	31	*3.1*
*Mother's education *												
Primary	11,953	*30.4*	445	*44.7*	273	*27.2*	9,158	*27.9*	532	*39.6*	235	*23.4*
Secondary	23,919	*60.9*	486	*48.8*	657	*65.4*	20,592	*62.6*	711	*52.9*	667	*66.4*
Tertiary	2,625	*6.7*	33	*3.3*	63	*6.3*	2,568	*7.8*	77	*5.7*	78	*7.8*
n.a.	759	*1.9*	31	*3.1*	12	*1.2*	560	*1.7*	23	*1.7*	24	*2.4*
*Mother's employment *												
No	21,767	*55.4*	619	*62.2*	484	*48.2*	16,449	*50.0*	766	*57.0*	411	*40.9*
Yes	16,847	*42.9*	357	*35.9*	515	*51.2*	15,937	*48.5*	557	*41.5*	579	*57.7*
n.a.	642	*1.6*	19	*1.9*	6	*0.6*	492	*1.5*	20	*1.5*	14	*1.4*
*Individual's education*												
Lower-secondary	17,108	*43.6*	485	*48.7*	414	*41.2*	9,655	*29.4*	455	*33.9*	291	*29.0*
Upper-secondary	18,924	*48.2*	392	*39.4*	442	*44.0*	18,703	*56.9*	606	*45.1*	471	*46.9*
Tertiary	3,224	*8.2*	118	*11.9*	149	*14.8*	4,520	*13.7*	282	*21.0*	242	*24.1*
*Student status *												
No	37,162	*94.7*	982	*98.7*	984	*97.9*	30,855	*93.8*	1,319	*98.2*	978	*97.4*
Yes	1,771	*4.5*	6	*0.6*	18	*1.8*	1,885	*5.7*	11	*0.8*	23	*2.3*
n.a.	323	*0.8*	7	*0.7*	3	*0.3*	138	*0.4*	13	*1.0*	3	*0.3*
*Having children*												
No	38,626	*98.4*	859	*86.3*	905	*90.0*	31,935	*97.1*	1,186	*88.3*	915	*91.1*
Yes	537	*1.4*	134	*13.5*	100	*10.0*	872	*2.7*	156	*11.6*	87	*8.7*
n.a.	93	*0.2*	2	*0.2*	0	*0.0*	71	*0.2*	1	*0.1*	2	*0.2*
*Left home for non-union-related reasons*												
No	30,339	*77.3*	708	*71.2*	517	*51.4*	25,772	*78.4*	1,140	*84.9*	558	*55.6*
Yes	8,917	*22.7*	287	*28.8*	488	*48.6*	7,106	*21.6*	203	*15.1*	446	*44.4*
*Calendar time*												
1995-1999	7,469	*19.0*	99	*9.9*	103	*10.2*	6,937	*21.1*	244	*18.2*	123	*12.3*
2000-2003	8,567	*21.8*	223	*22.4*	190	*18.9*	7,521	*22.9*	340	*25.3*	181	*18.0*
2004-2007	9,063	*23.1*	259	*26.0*	242	*24.1*	7,667	*23.3*	299	*22.3*	253	*25.2*
2008-2011	7,940	*20.2*	222	*22.3*	277	*27.6*	6,209	*18.9*	301	*22.4*	266	*26.5*
2012-2015	6,217	*15.8*	192	*19.3*	193	*19.2*	4,544	*13.8*	159	*11.8*	181	*18.0*
*Area of residence*												
North	15,533	*39.6*	323	*32.5*	622	*61.9*	13,297	*40.4*	449	*33.4*	613	*61.1*
Centre	6,792	*17.3*	153	*15.4*	195	*19.4*	5,397	*16.4*	225	*16.8*	170	*16.9*
South/Isles	16,931	*43.1*	519	*52.2*	188	*18.7*	14,184	*43.1*	669	*49.8*	221	*22.0*

**Table 3:** Results from discrete-time competing risks models of the transition into first marriage and first cohabitation: relative risk ratios (RRR), men

	MARRIAGE											COHABITATION					
	Mod. 1			Mod. 2				Mod. 3				Mod. 1		Mod. 2		Mod. 3	
	RRR	*P>z*		RRR		*P>z*		RRR		*P>z*		RRR	*P>z*	RRR	*P>z*	RRR	*P>z*
*Individual’s age (ref.: 25-29)*																	
16–20	0.075	*0.000*		0.072		*0.000*		0.072		*0.000*		0.397	*0.000*	0.374	*0.000*	0.367	*0.000*
21–24	0.353	*0.000*		0.347		*0.000*		0.347		*0.000*		0.582	*0.000*	0.577	*0.000*	0.571	*0.000*
30–34	1.355	*0.000*		1.479		*0.000*		1.479		*0.000*		0.948	*0.551*	0.937	*0.482*	0.946	*0.544*
35+	0.701	*0.005*		0.899		*0.442*		0.898		*0.436*		0.614	*0.000*	0.620	*0.001*	0.643	*0.003*
*Employment (ref.: permanent)*																	
Self-employment	1.016	*0.857*		0.950		*0.557*		0.949		*0.553*		0.842	*0.069*	0.936	*0.488*	0.941	*0.527*
Temporary	0.706	*0.001*		0.667		*0.000*		0.667		*0.000*		0.813	*0.027*	0.896	*0.243*	0.904	*0.284*
No work	0.360	*0.000*		0.315		*0.000*		0.314		*0.000*		0.332	*0.000*	0.426	*0.000*	0.432	*0.000*
*Parental separation (Ref.: no)*																	
Yes	0.731	*0.079*		0.805		*0.229*		0.802		*0.222*		1.417	*0.003*	1.288	*0.032*	1.317	*0.020*
*Mother’s education (ref.: primary)*																	
Secondary	0.716	*0.000*		0.761		*0.000*		0.762		*0.000*		1.297	*0.001*	1.244	*0.007*	1.241	*0.007*
Tertiary	0.589	*0.007*		0.645		*0.027*		0.645		*0.027*		1.078	*0.631*	1.022	*0.890*	1.026	*0.870*
*Mother’s employment (ref.: No)*																	
Yes	0.972	*0.697*		1.046		*0.538*		1.048		*0.519*		1.361	*0.000*	1.252	*0.001*	1.243	*0.002*
*Individual’s education (ref.: lower-secondary) *																	
Upper-secondary	0.797	*0.002*		0.782		*0.001*		0.780		*0.001*		0.828	*0.011*	0.857	*0.038*	0.863	*0.048*
Tertiary	0.834	*0.115*		0.829		*0.108*		0.828		*0.105*		0.896	*0.317*	0.903	*0.355*	0.902	*0.349*
*Student status (ref.: no)*																	
Yes	0.359	*0.014*		0.384		*0.021*		0.383		*0.021*		0.664	*0.095*	0.668	*0.103*	0.682	*0.121*
*Have children (ref. no)*																	
Yes	8.051	*0.000*		8.267		*0.000*		8.246		*0.000*		6.696	*0.000*	6.749	*0.000*	6.848	*0.000*
*Left home for non-union related reasons (ref.: no)*																	
Yes	0.950	*0.496*		0.992		*0.912*		0.991		*0.907*		2.522	*0.000*	2.438	*0.000*	2.425	*0.000*
*Calendar time (ref.: 2004-2007)*																	
1995-1999				1.035		*0.792*		1.047		*0.772*				0.758	*0.027*	0.942	*0.688*
2000-2003				1.181		*0.092*		1.224		*0.194*				0.904	*0.319*	1.072	*0.653*
2008-2011				0.825		*0.047*		0.839		*0.105*				1.141	*0.154*	1.243	*0.036*
2012-2015				0.717		*0.002*		0.696		*0.004*				0.833	*0.085*	1.042	*0.749*
*Area of residence (ref.: North)*																	
Centre				1.117		*0.276*		1.104		*0.353*				0.735	*0.000*	0.812	*0.022*
South/Isles				1.706		*0.000*		1.625		*0.001*				0.347	*0.000*	0.516	*0.000*
*Unemployment rate (15-24)*								1.121		*0.685*						0.381	*0.002*
*CCCI*								0.938		*0.739*						0.774	*0.169*
Constant	0.072	*0.000*		0.055		*0.000*		0.055		*0.000*		0.028	*0.000*	0.042	*0.000*	0.049	*0.000*

**Table 4:** Results from discrete-time competing risks models of the transition into first marriage and first cohabitation: relative risk ratios (RRR), women

	MARRIAGE											COHABITATION					
	Mod. 1			Mod. 2				Mod. 3				Mod. 1		Mod. 2		Mod. 3	
	RRR	*P>z*		RRR		*P>z*		RRR		*P>z*		RRR	*P>z*	RRR	*P>z*	RRR	*P>z*
*Individual’s age (ref.: 25-29)*																	
16–20	0.153	*0.000*		0.143		*0.000*		0.144		*0.000*		0.468	*0.000*	0.474	*0.000*	0.473	*0.000*
21–24	0.497	*0.000*		0.476		*0.000*		0.480		*0.000*		0.706	*0.000*	0.715	*0.000*	0.714	*0.000*
30–34	0.827	*0.027*		0.894		*0.204*		0.897		*0.218*		0.855	*0.113*	0.810	*0.037*	0.810	*0.037*
35+	0.348	*0.000*		0.446		*0.000*		0.447		*0.000*		0.338	*0.000*	0.310	*0.000*	0.314	*0.000*
*Employment (ref.: permanent)*																	
Self-employment	1.023	*0.846*		0.975		*0.825*		0.976		*0.838*		0.726	*0.015*	0.771	*0.050*	0.779	*0.061*
Temporary	0.765	*0.004*		0.747		*0.002*		0.747		*0.002*		0.848	*0.064*	0.900	*0.243*	0.902	*0.254*
No work	0.742	*0.000*		0.649		*0.000*		0.651		*0.000*		0.350	*0.000*	0.429	*0.000*	0.433	*0.000*
*Parental separation (ref.: no)*																	
Yes	0.568	*0.000*		0.595		*0.001*		0.594		*0.001*		1.736	*0.000*	1.652	*0.000*	1.662	*0.000*
*Mother’s education (ref.: primary)*																	
Secondary	0.803	*0.001*		0.873		*0.039*		0.872		*0.037*		1.297	*0.002*	1.178	*0.052*	1.177	*0.053*
Tertiary	0.830	*0.169*		0.876		*0.335*		0.875		*0.328*		1.208	*0.196*	1.115	*0.460*	1.120	*0.440*
*Mother’s employment (ref.: no)*																	
Yes	0.865	*0.016*		0.913		*0.135*		0.911		*0.126*		1.309	*0.000*	1.221	*0.004*	1.212	*0.006*
*Individual’s education (ref.: lower-secondary)*																	
Upper-secondary	0.732	*0.000*		0.721		*0.000*		0.719		*0.000*		0.708	*0.000*	0.715	*0.000*	0.712	*0.000*
Tertiary	1.024	*0.795*		1.058		*0.539*		1.057		*0.544*		0.822	*0.061*	0.819	*0.056*	0.817	*0.053*
*Student status (ref.: no)*																	
Yes	0.214	*0.000*		0.246		*0.000*		0.245		*0.000*		0.483	*0.001*	0.487	*0.001*	0.488	*0.001*
*Have children (ref.: no)*																	
Yes	4.635	*0.000*		4.953		*0.000*		4.955		*0.000*		2.654	*0.000*	2.422	*0.000*	2.415	*0.000*
*Left home for non-union related reasons (ref.: no)*																	
Yes	0.455	*0.000*		0.479		*0.000*		0.478		*0.000*		2.126	*0.000*	2.029	*0.000*	2.015	*0.000*
*Calendar time (ref.: 2004-2007)*																	
1995-1999				1.315		*0.004*		1.185		*0.155*				0.697	*0.002*	0.692	*0.009*
2000-2003				1.260		*0.006*		1.045		*0.739*				0.793	*0.022*	0.708	*0.025*
2008-2011				1.117		*0.199*		1.034		*0.728*				1.180	*0.073*	1.139	*0.211*
2012-2015				0.662		*0.000*		0.691		*0.002*				0.930	*0.496*	1.072	*0.587*
*Area of residence (ref.: North***)**																	
Centre				1.272		*0.005*		1.294		*0.004*				0.822	*0.030*	0.875	*0.166*
South/Isles				1.543		*0.000*		1.631		*0.000*				0.493	*0.000*	0.614	*0.001*
*Unemployment rate (15-24)*								0.872		*0.563*						0.578	*0.058*
*CCCI*								1.356		*0.065*						1.213	*0.300*
Constant	0.150	*0.000*		0.107		*0.000*		0.100		*0.000*		0.045	*0.000*	0.065	*0.000*	0.066	*0.000*

**Table 5:** Robustness checks: relative risk ratios (RRR) from discrete-time competing risks models of the transition into first marriage and first cohabitation with different specifications of the macro-level variables, men

	MARRIAGE						COHABITATION					
	Mod. 4		Mod. 5		Mod. 6		Mod. 4		Mod. 5		Mod. 6	
	RRR	*P>z*	RRR	*P>z*	RRR	*P>z*	RRR	*P>z*	RRR	*P>z*	RRR	*P>z*
*Individual’s age (ref.: 25-29)*												
16–20	0.077	*0.000*	0.071	*0.000*	0.071	0.000	0.353	*0.000*	0.367	*0.000*	0.368	*0.000*
21–24	0.357	*0.000*	0.340	*0.000*	0.344	0.000	0.560	*0.000*	0.572	*0.000*	0.572	*0.000*
30–34	1.385	*0.000*	1.460	*0.000*	1.492	0.000	0.980	*0.822*	0.958	*0.641*	0.944	*0.530*
35+	0.754	*0.030*	0.850	*0.228*	0.910	0.499	0.683	*0.007*	0.650	*0.003*	0.642	*0.002*
*Employment (ref.: permanent)*												
Self-employment	0.952	*0.577*	0.958	*0.624*	0.957	0.614	0.942	*0.530*	0.939	*0.509*	0.937	*0.497*
Temporary	0.658	*0.000*	0.667	*0.000*	0.671	0.000	0.912	*0.330*	0.908	*0.307*	0.903	*0.282*
No work	0.317	*0.000*	0.314	*0.000*	0.313	0.000	0.430	*0.000*	0.433	*0.000*	0.433	*0.000*
*Parental separation (ref.: no)*												
Yes	0.781	*0.171*	0.793	*0.201*	0.802	0.224	1.334	*0.015*	1.327	*0.016*	1.317	*0.020*
*Mother’s education (ref.: primary)*												
Secondary	0.732	*0.000*	0.762	*0.000*	0.770	0.000	1.264	*0.003*	1.239	*0.007*	1.237	*0.008*
Tertiary	0.615	*0.014*	0.654	*0.032*	0.660	0.036	1.053	*0.741*	1.023	*0.887*	1.018	*0.909*
*Mother’s employment (ref.: no)*												
Yes	1.032	*0.669*	1.056	*0.458*	1.060	0.424	1.249	*0.001*	1.233	*0.002*	1.235	*0.002*
*Individual’s education (ref.: lower-secondary)*												
Upper-secondary	0.788	*0.001*	0.782	*0.001*	0.780	0.001	0.863	*0.048*	0.864	*0.050*	0.862	*0.047*
Tertiary	0.832	*0.112*	0.829	*0.108*	0.826	0.101	0.903	*0.354*	0.902	*0.349*	0.903	*0.350*
*Student status (ref.: no)*												
Yes	0.352	*0.012*	0.370	*0.017*	0.381	0.020	0.710	*0.163*	0.689	*0.130*	0.683	*0.122*
*Having children (ref.: no)*												
Yes	8.190	*0.000*	8.236	*0.000*	8.237	0.000	6.858	*0.000*	6.869	*0.000*	6.854	*0.000*
*Left home for non-union related reasons (ref.: no)*												
Yes	0.982	*0.804*	1.000	*0.995*	1.001	0.991	2.428	*0.000*	2.416	*0.000*	2.419	*0.000*
*Calendar time (ref. 2004-2007)*												
1995-1999					0.948	0.752					1.016	*0.921*
2000-2003					1.132	0.437					1.119	*0.477*
2008-2011					0.873	0.227					1.201	*0.098*
2012-2015					0.789	0.098					0.960	*0.794*
*Area of residence (ref.: North)*												
Centre	1.140	*0.211*	1.035	*0.768*	1.075	0.557	0.822	*0.028*	0.872	*0.164*	0.843	*0.101*
South/Isles	1.823	*0.000*	1.207	*0.252*	1.288	0.189	0.546	*0.000*	0.668	*0.014*	0.597	*0.007*
Unemployment rate (15-24)	0.827	*0.401*	0.995	*0.981*	1.151	0.619	0.339	*0.000*	0.295	*0.000*	0.361	*0.001*
CCCI	1.257	*0.052*	1.104	*0.416*	0.974	0.893	0.821	*0.087*	0.875	*0.269*	0.759	*0.139*
Cohabitation diffusion			0.972	*0.035*	0.988	0.470			1.016	*0.159*	1.012	*0.453*
Secularisation			0.987	*0.018*	0.986	0.015			1.003	*0.569*	1.003	*0.546*
Constant	0.050	*0.000*	0.131	*0.000*	0.121	0.000	0.051	*0.000*	0.038	*0.000*	0.038	*0.000*

**Table 6:** Robustness checks: relative risk ratios (RRR) from discrete-time competing risks models of the transition into first marriage and first cohabitation with different specifications of the macro-level variables, women

	MARRIAGE						COHABITATION					
	Mod. 4		Mod. 5		Mod. 6		Mod. 4		Mod. 5		Mod. 6	
	RRR	*P>z*	RRR	*P>z*	RRR	*P>z*	RRR	*P>z*	RRR	*P>z*	RRR	*P>z*
*Individual’s age (ref.: 25-29)*												
16–20	0.155	*0.000*	0.146	*0.000*	0.143	*0.000*	0.435	*0.000*	0.462	*0.000*	0.472	*0.000*
21–24	0.504	*0.000*	0.486	*0.000*	0.477	*0.000*	0.682	*0.000*	0.705	*0.000*	0.714	*0.000*
30–34	0.873	*0.118*	0.908	*0.271*	0.902	*0.246*	0.863	*0.142*	0.834	*0.072*	0.810	*0.037*
35+	0.399	*0.000*	0.448	*0.000*	0.455	*0.000*	0.352	*0.000*	0.314	*0.000*	0.310	*0.000*
*Employment (ref.: permanent)*												
Self-employment	0.977	*0.841*	0.996	*0.973*	0.994	*0.958*	0.783	*0.066*	0.777	*0.059*	0.777	*0.059*
Temporary	0.734	*0.001*	0.753	*0.003*	0.756	*0.003*	0.923	*0.370*	0.903	*0.258*	0.900	*0.245*
No work	0.657	*0.000*	0.652	*0.000*	0.649	*0.000*	0.430	*0.000*	0.433	*0.000*	0.433	*0.000*
*Parental separation (ref.: no)*												
Yes	0.586	*0.001*	0.594	*0.001*	0.597	*0.001*	1.694	*0.000*	1.672	*0.000*	1.660	*0.000*
*Mother’s education (ref.: primary)*												
Secondary	0.847	*0.010*	0.870	*0.033*	0.872	*0.037*	1.246	*0.008*	1.203	*0.028*	1.176	*0.054*
Tertiary	0.858	*0.260*	0.896	*0.421*	0.893	*0.406*	1.196	*0.221*	1.151	*0.340*	1.119	*0.445*
*Mother’s employment (ref.: no)*												
Yes	0.902	*0.092*	0.921	*0.178*	0.924	*0.195*	1.213	*0.006*	1.194	*0.011*	1.206	*0.008*
*Individual’s education (ref.: lower-secondary)*												
Upper-secondary	0.717	*0.000*	0.714	*0.000*	0.715	*0.000*	0.714	*0.000*	0.716	*0.000*	0.714	*0.000*
Tertiary	1.036	*0.696*	1.040	*0.668*	1.046	*0.623*	0.838	*0.091*	0.824	*0.064*	0.818	*0.055*
*Student status (ref.: no)*												
Yes	0.227	*0.000*	0.237	*0.000*	0.243	*0.000*	0.524	*0.003*	0.499	*0.001*	0.489	*0.001*
*Having children (ref.: no)*												
Yes	4.852	*0.000*	4.988	*0.000*	5.006	*0.000*	2.448	*0.000*	2.392	*0.000*	2.402	*0.000*
*Left home for non-union related reasons (ref.: no)*												
Yes	0.474	*0.000*	0.479	*0.000*	0.479	*0.000*	2.030	*0.000*	2.007	*0.000*	2.010	*0.000*
*Calendar time (ref. 2004-2007)*												
1995-1999					1.069	*0.607*					0.758	*0.076*
2000-2003					0.959	*0.763*					0.748	*0.068*
2008-2011					1.077	*0.455*					1.084	*0.464*
2012-2015					0.781	*0.067*					0.948	*0.733*
*Area of residence (ref.: North)*												
Centre	1.336	*0.001*	1.261	*0.019*	1.270	*0.021*	0.891	*0.218*	1.016	*0.875*	0.940	*0.569*
South/Isles	1.808	*0.000*	1.340	*0.026*	1.312	*0.083*	0.663	*0.001*	0.913	*0.558*	0.716	*0.069*
Unemployment rate (15-24)	0.645	*0.027*	0.723	*0.106*	0.904	*0.673*	0.516	*0.004*	0.436	*0.001*	0.581	*0.066*
CCCI	1.625	*0.000*	1.444	*0.000*	1.420	*0.035*	0.843	*0.138*	0.957	*0.714*	1.183	*0.369*
Cohabitation diffusion			0.977	*0.048*	0.987	*0.400*			1.037	*0.001*	1.022	*0.166*
Secularisation			0.988	*0.015*	0.987	*0.009*			0.998	*0.696*	0.998	*0.678*
Constant	0.098	*0.000*	0.222	*0.000*	0.208	*0.000*	0.070	*0.000*	0.053	*0.000*	0.060	*0.000*

## Footnotes

1 We excluded the 10 cases reporting the first co-residential union before 16 as either unrealistic or outliers.

2 According to Italian labour market characteristics, this category includes entrepreneurs and professionals, who tend to have favourable characteristics in the labour market similar to permanent workers, but also independent workers and freelancers (self-employed working on their own account), who may suffer job precariousness.

3 The temporal dimension indicates precariousness, though situations clearly differ depending on the contract duration (which can span from several months to 2–3 years). In Italy, fixed-term contracts generally offer the same rights and social protection guarantees as permanent employment. However, casual work does not entail a dependent relationship between worker and employer. Instead, it often involves a ready supply of cheap and occasional labour, typically for specific and time-limited tasks or projects, without rights to sickness absence or annual leave. Moreover, their contracts are generally of shorter duration than temporary ones (for an extended discussion, see Pirani, 2017). Based on preliminary analyses that distinguished between the two subcategories but revealed no substantial variations in the results, we decided, for the sake of simplicity, to collapse them into one category.

4 Due to data limitations, we are not able to distinguish between inactive and unemployed individuals.

5 The current consumer confidence index (CCCI) is elaborated based on respondent assessments of the Italian general economic situation, household financial situation, family budget, savings opportunities and opportunities for durable goods purchases. The CCCI is expressed as index numbers based on 2010 = 100.

6 For both macro-level indicators, we also tested a lag of 2 years, but the results were virtually unchanged. We thus opted to utilise the 1-year lag specification, also considering that the CCCI was available only since 1995.

7 Both the cohabitation diffusion and secularisation indicators have been computed using the annual Italian cross-sectional surveys “Aspects of Daily Life” conducted by ISTAT since 1993. Specifically, starting from micro-level data, we computed the indicator of cohabitation diffusion as the proportion of cohabiting couples on the total number of couples aged 25–49, for a given year in a given Italian region. Similarly, the indicator of secularisation has been computed, again at the year-region level, as the percentage of people declaring to never or rarely attend religious services.
